# Supplementary material for: 5,6-dimethylxanthenone-4-acetic acid (DMXAA), a partial STING agonist, competes for human STING activation
Source: Front Immunol. 2024 Mar 12;15:1353336. doi: 10.3389/fimmu.2024.1353336 (PMC10963404; doi:10.3389/fimmu.2024.1353336)
Supplement: Supplementary file 1 [file DataSheet_1.pdf]

Supplementary Material

5,6-dimethylxanthenone-4-acetic acid (DMXAA), a Partial STING Agonist, Competes for Human STING Activation

Burcu Temizoz[†], Takayuki Shibahara[†], Kou Hioki, Tomoya Hayashi, Kouji Kobiyama, Michelle Sue Jann Lee, Naz Surucu, Erdal Sag, Atsushi Kumanogoh, Masahiro Yamamoto, Mayda Gursel, Seza Ozen, Etsushi Kuroda, Cevayir Coban, and Ken J. Ishii*

†These authors contributed equally to this work and share first authorship

*** Correspondence:**

Ken J. Ishii (K.J.I.)

kenishii@ims.u-tokyo.ac.jp

Supplementary Figures

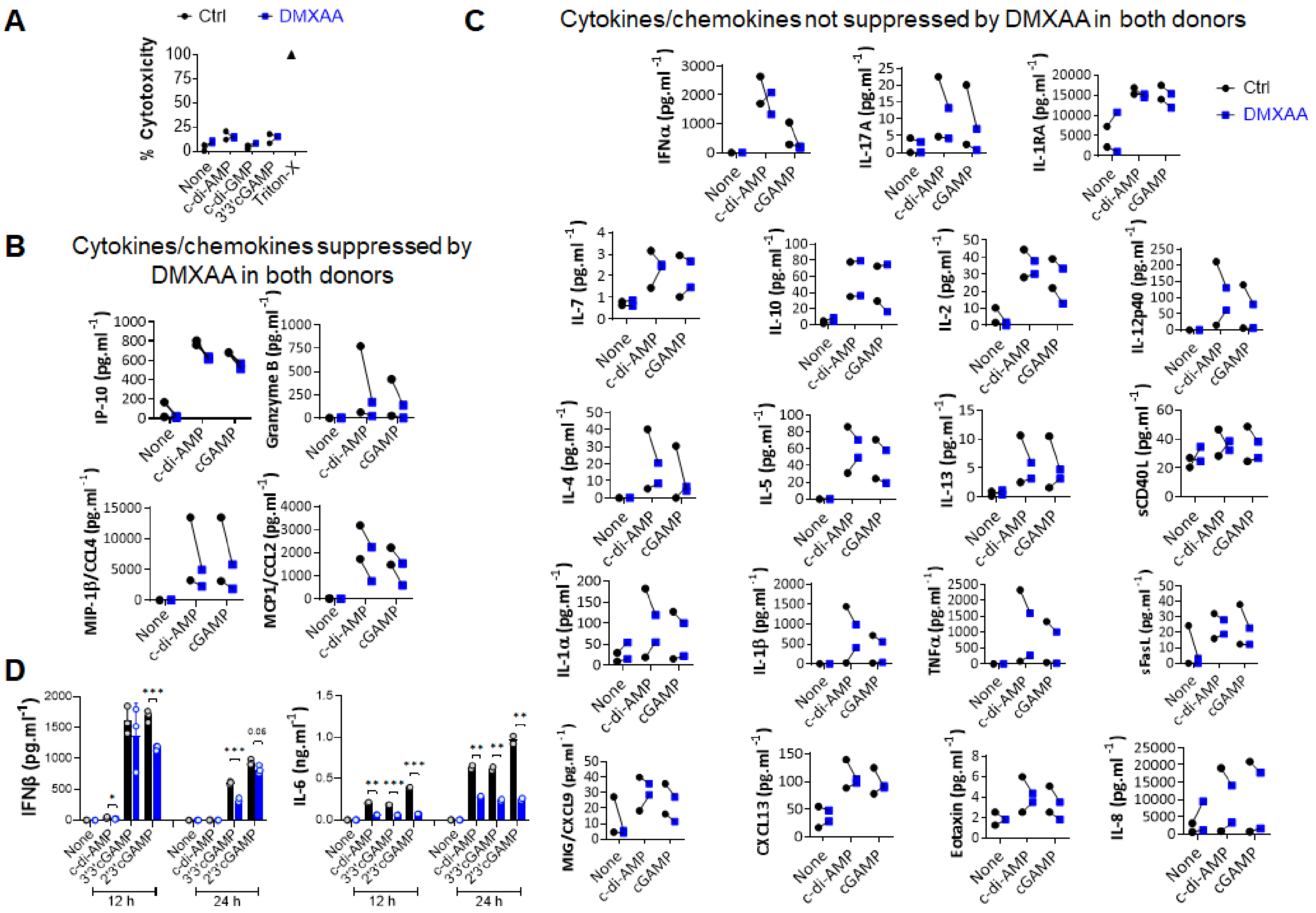

Supplementary Figure 1. Fresh human PBMCs from two healthy donors were stimulated with the indicated CDNs (10 µg/ml) for 24 h after 90 minutes of DMXAA (100 µg/ml) pretreatment. **(A)** Cell death was measured using lactate dehydrogenase (LDH) release assay in two of the PBMC donors. Data are shown as scatter plots showing individual values from each donor. **(B.C)** Cytokine production was measured using the human 25-plex Bio-plex Cytokine Assay. **(B)** IP-10, granzyme B, MIP-1b/CCL4, MCP1/CCL2 and **(C)** eotaxin, CXCL13, IL-1a, IL-1b, IL1-RA, IL-2, IL-4, IL-5, IL-6, IL-7, IL-8, IL-9, IL-10, IL-12p40, IL-13, IL-15, IL-17A, MIP-1a, MIG, MCP-1, TNFa, sCD40L

and sFasL levels from Bio-plex analysis are shown as scatter plots showing individual values from each donor. **(D)** PMA-differentiated THP1 dual reporter cells were pretreated with DMXAA (100 μg/ml) for 90 min and stimulated with the indicated CDNs (10 μg/ml) for 12 or 24 h in three independent experiments. The levels of type I IFN and IL-6 levels in the supernatants were measured using ELISA. Data are shown as the mean ± SD of triplicates for IFNb and duplicates for IL-6 (*p < 0.05, **p < 0.01, ***p < 0.001, Student's t test).

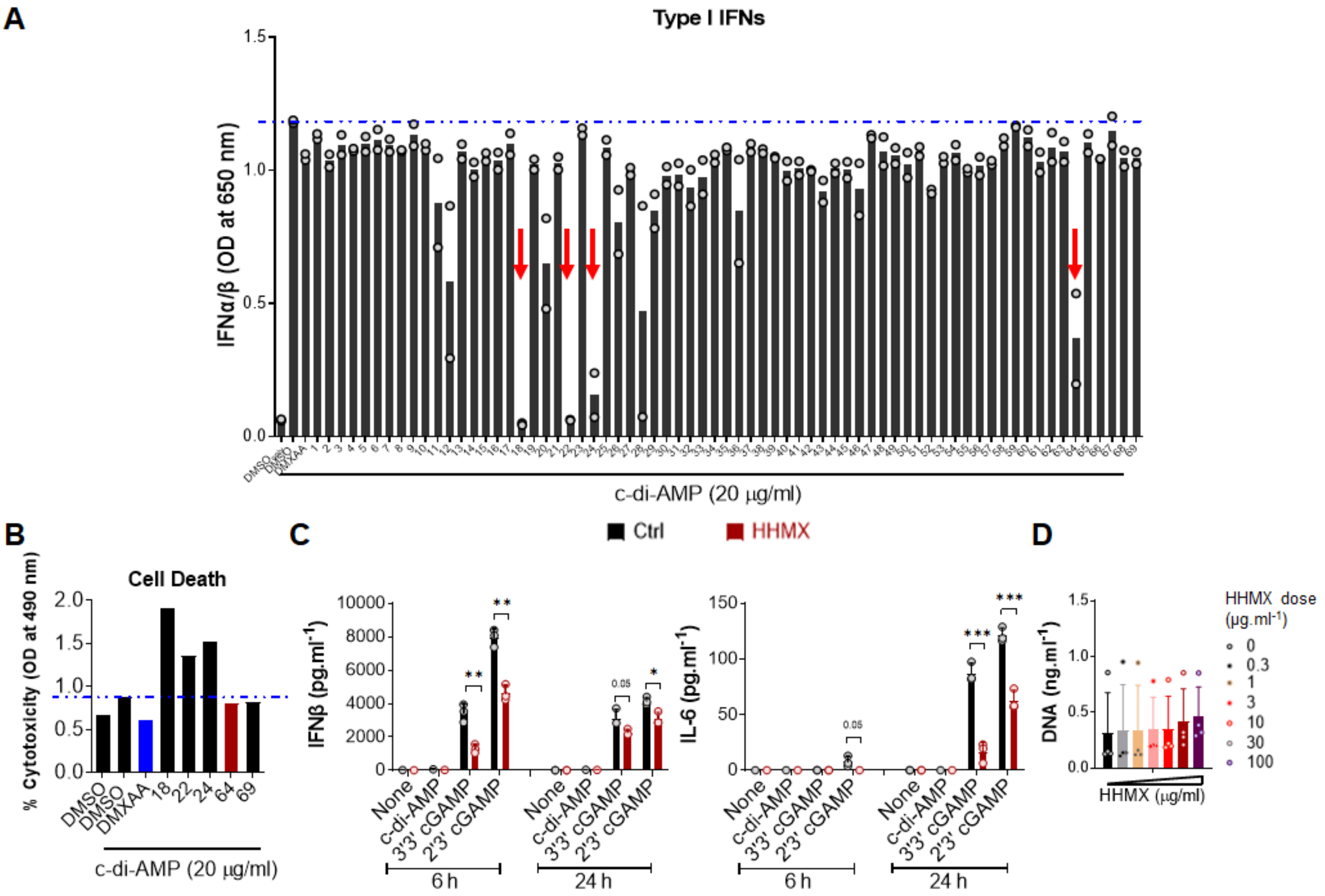

Supplementary Figure 2: (A) Fresh human PBMCs from two healthy donors were stimulated with c-di-AMP (20 μg/ml) with or without DMXAA or its derivatives (100 μg/ml) for 24 h. The levels of type I IFN in the supernatants were measured using HEK-Blue IFNα/β reporter cells. Bar graphs showing individual data and mean are plotted. Red arrows represent DMXAA derivatives with robust suppressive effect on STING-induced type I IFN production in human PBMCs. **(B)** Cell

death was measured using the lactate dehydrogenase (LDH) release assay. Representative data from one PBMC donor among two different donors are shown as bar graph. **(C)** PMA-differentiated THP1 dual reporter cells were stimulated with HHMX (30 µg/ml) together with the indicated CDNs (10 µg/ml) for 6 or 24 h. The levels of type I IFN and IL-6 levels in the supernatants were measured using ELISA. Data are shown as the mean ± SD of triplicates (*p < 0.05, **p < 0.01, ***p < 0.001, Student's t test). **(D)** PMA-differentiated THP1 dual reporter cells were stimulated with the indicated concentrations of HHMX (0, 0.3, 1, 3, 10, 30, 100 µg/ml) 24 h. DNA release was measured using the QuantiFluor dsDNA detection kit (Promega) according to manufacturer's instructions. Data are shown as the mean ± SD from four independent experiments (n=4).

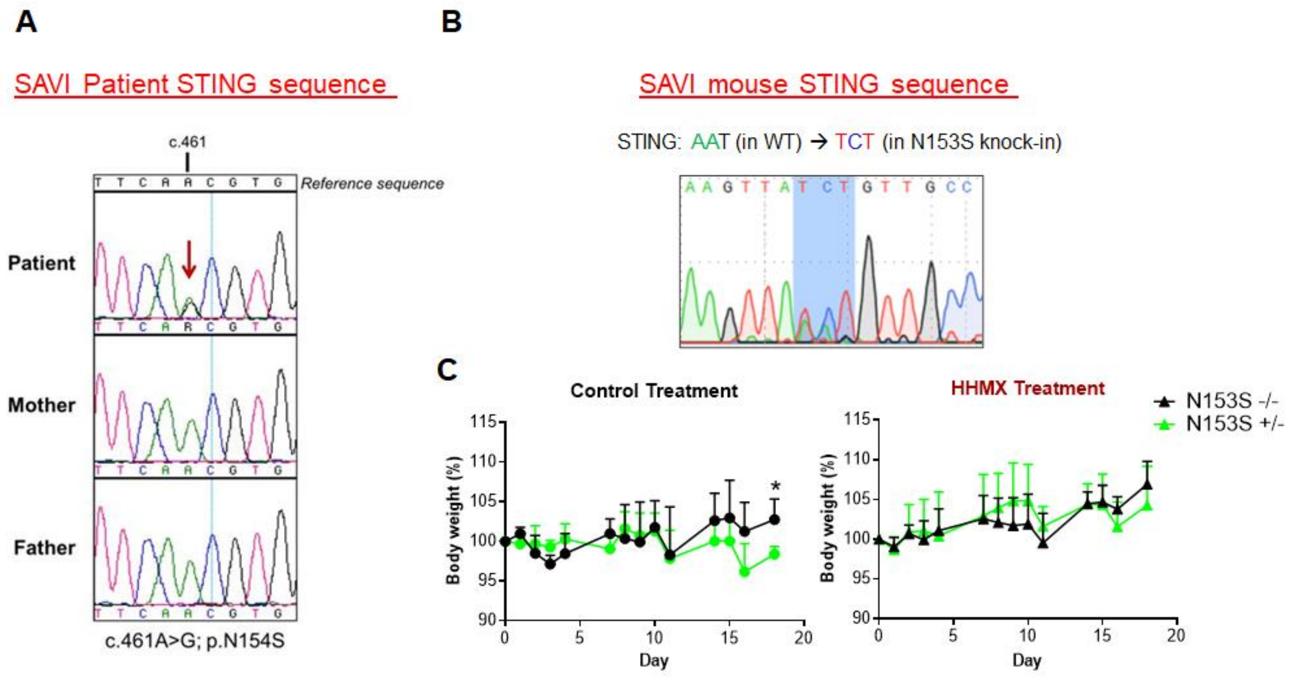

Supplementary Figure 3: (A) Sequencing data of STING in SAVI patients. **(B)** Sequencing data of STING in SAVI mouse. **(C)** Eight–to-thirteen-week-old N153S+/- SAVI mice (n=4-6) and their WT littermate (n=3-6) controls were orally administered 500 µg of HHMX or control, respectively, for 3 weeks every weekday (5 times/week). Body weight and symptoms of mice were

monitored until the day of sacrifice. Body weight percentages of the mice over the period of 18 days are plotted as line graph for control and HHMX treatment groups as mean (*$p < 0.05$, Student's t test).
